# Pre-Exercise Caffeine Intake Attenuates the Negative Effects of Ramadan Fasting on Several Aspects of High-Intensity Short-Term Maximal Performances in Adolescent Female Handball Players

**DOI:** 10.3390/nu15153432

**Published:** 2023-08-03

**Authors:** Houda Bougrine, Nidhal Nasser, Raouf Abdessalem, Achraf Ammar, Hamdi Chtourou, Nizar Souissi

**Affiliations:** 1 Physical Activity Research Unit, Sport and Health (UR18JS01), National Observatory of Sports, Tunis 1003, Tunisia; houdabougrine@live.fr (H.B.); nassernidhal@hotmail.com (N.N.); raoufabdesalem18@gmail.com (R.A.); h_chtourou@yahoo.fr (H.C.); n_souissi@yahoo.fr (N.S.); 2 High Institute of Sport and Physical Education Ksar-Saïd, Manouba University, Mannouba 2010, Tunisia; 3 High Institute of Sport and Physical Education Sfax, University of Sfax, Sfax 3000, Tunisia; 4 Department of Training and Movement Science, Institute of Sport Science, Johannes Gutenberg-University Mainz, 55099 Mainz, Germany; 5 Research Laboratory, Molecular Bases of Human Pathology, LR19ES13, Faculty of Medicine of Sfax, University of Sfax, Sfax 3029, Tunisia

**Keywords:** Ramadan intermittent fasting, caffeine time of the day, physical performance, sleep quality, female athletes

## Abstract

The aim of this investigation was to determine whether, after Ramadan, pre-exercise caffeine intake can reduce any possible negative effects of this month on short-term maximal performances in young female handball players. A randomized study involved thirteen young female handball players. Participants performed a squat jump (SJ), Illinois agility test (AG), and 5 m run shuttles test (total (TD) and peak (PD) distances) at 08:00 AM and 06:00 PM on three different occasions: one week before Ramadan (Pre-R), the last week of Ramadan (R), and the week after Ramadan (Post-R). A placebo (Pla) or caffeine (Caff) (6 mg·kg^−1^) was administered 60 min before exercise test sessions at two distinct times of day (08:00 AM and 06:00 PM) during the two periods: Pre and Post-R. The PSQI and dietary intake were assessed during all testing periods. The results revealed that Pre-R, (SJ, AG, TD, and PD) test performances were greater in the evening (PM) than in the morning (AM) (all *p* < 0.001). However, compared with Pre-R, PM performances declined significantly during R (all *p* < 0.001) and Post-R (*p* < 0.05, *p* < 0.01, *p* < 0.01 and *p* < 0.001, respectively). In addition, Pre-R, AM Caff produced moderate significant improvements compared with AM Pla, with small-to-no beneficial effects observed with PM Caff in SJ (4.8% vs. 1%), AG (1.8% vs. 0.8%), TD (2.8% vs. 0.3%), and PD (6% vs. 0.9%). Nevertheless, Caff produced moderate ergogenic effects during both AM and PM sessions during Post-R in SJ (4.4% vs. 2.4%), AG (1.7% vs. 1.5%), TD (2.9% vs. 1.3%), and PD (5.8% vs. 3%) with values approaching those of Pre-R Pla within the same time of day (*p* > 0.05, *p* > 0.05, *p* < 0.05, and *p* < 0.05, respectively). In summary, pre-exercise Caff intake with a dose equivalent to 6 mg·kg^−1^ reduced the negative effects of Ramadan fasting in several aspects of short-term maximal performances in young female handball players at both times of the day.

## 1. Introduction

Fasting is advocated in almost all religions [1], Ramadan fasting, which is one of the five pillars of Islam and is observed by over 1.6 billion people worldwide, is a familiar practice for Muslims. During that month (28–30 consecutive days), the pubertal healthy adults and adolescents abstained from taking any fluid, food, cigarettes, medications, or engaging in sexual intercourse from dawn until sunset [2]. Since this Islamic month follows the lunar cycle, it can be observed at various periods of the year (in different seasons). In recent years, preparatory phases, training, or international competitions of handball and numerous other team ball sports scheduled all across the calendar year have been known to intersect the fasting month of Ramadan.

Evidence from this month’s modifications has incited numerous researchers to examine the impact of Ramadan fasting on athletes’ physical performance, though the findings remain equivocal. In this context, previous studies have reported impairments of high-intensity short-term maximal physical performances for both male and female athletes during jump trials [2,3,4], agility tests [2,4], and repeated sprint exercises [2,5] during and immediately following Ramadan. Other investigations, however, have reported that jump performance [6,7,8], agility tests [8,9,10,11], and repeated sprint exercises [12], remained unchanged during and after Ramadan relative to before Ramadan. In order to optimize athletic performance, numerous strategies have been proposed to adapt to the training demands during Ramadan fasting, such as managing poor sleep by adjusting the training schedule [13], managing training patterns [14], adjusting nighttime meals as well as fluid intake [15], and mouth-rinsing with different stimulant substances [16,17,18]. However, no specific strategies and techniques have been proposed to meet the requirements of training and national and/or international competitions when matches, tournaments, or preparations are scheduled immediately following Ramadan. While it has been clearly stated that changes in sleep and diet [19,20] seem to be crucial determinants in enhancing competition performance recovery [21,22] and that Ramadan fasting can impair sleep duration and cause a reduction in sleep quality, especially during the last days of Ramadan, which can continue shortly after Ramadan to return to initial values of Pre-Ramadan [2,23], the topic has not yet been thoroughly evaluated during this period.

Although it is known that poor sleep quality and duration can negatively affect mood and cognitive performance [24] as well as physical functions such as restoration [25] and short-term maximal performance [26], the strategy for dealing with this impairment during this period has been little investigated. Previous research, on the other hand, found that caffeine consumption can mitigate the negative effects of sleep deprivation on both cognitive and physical performance [27,28]. Caffeine consumption has thus been demonstrated to be useful for both genders of athletes when examined during team sports [29,30]. In fact, caffeine impacts the body to enhance exercise performance by blocking central and peripheral adenosine receptors, diminishing adenosine’s effects on neurotransmission, and promoting arousal and exercise performance [31]. Surprisingly, there is a lack of evidence regarding the impact of caffeine supplementation at different times of the day, which is especially pronounced in team ball athletes, particularly young female athletes. In this regard, only one recent study [32] investigated whether the ergogenic benefits of caffeine administration differed depending on the time of day in this young female sample of team ball athletes. This common ergogenic aid, according to this study, aids in mitigating the morning decline in both cognitive and physical performance [32]. To date, no study has explored the effectiveness of caffeine intake during the Post-Ramadan fasting period. However, it would be interesting to examine whether caffeine would be effective at all times of the day to counteract the possible impairment of short-term maximal physical exercise caused by cumulative fatigue, dietary changes, and sleep patterns during the Ramadan fasting month.

Thus, the purpose of this study was to analyze the effects of pre-exercise caffeine ingestion immediately following Ramadan month (during the week Post-Ramadan) on short-term high-intensity performances in adolescent female handball players at various times of the day (morning and evening). Considering the published data, we hypothesized that caffeine intake can improve the short-term high-intensity performances of young female handball players during this period, with greater enhancement during the morning.

## 2. Materials and Methods

### 2.1. Participants

The minimum required sample size was calculated using the software G*power (version 3.1.9.6; Kiel University, Kiel, Germany). The F test family (repeated measures, within factors) was selected with three periods (Pre-Ramadan, last week of Ramadan, and Post-Ramadan), two times of day (Morning and evening), and two supplementation conditions (Caff and placebo). Based on physical performance data from Bougrine et al. [2,32], a sample size of 12 subjects has been identified to detect significant differences (effect size = 0.5, and α = 0.05) with an actual power of 95.23%. Out of the 37 reviewed surveys, 20 female handball players, competing in the first level of the Tunisian National League, were considered eligible and voluntarily participated in the study. During the experimental period, seven players dropped out due to logistical (02 players), injury (01 player), and methodological (fasting and non-fasting states and/or menstrual cycle phases: 04 players) issues. Data from the 13 participants who completed all experimental sessions has been analyzed, as depicted in Table 1. Athletes and parents received information about the experimental procedure, timing, types of exercises, and assessments they would need to complete before signing the written informed consent form. The Local Research Committee authorized all protocols and procedures, which were followed in accordance with the Declaration of Helsinki [33]. The following were the inclusion criteria: (a) being under 18 years old; (b) being light-Caff consumers (less than 100 mg/day); (c) and having previous experience in handball training (i.e., at least 2 years with a frequency of two or more training sessions per week). The exclusion criteria were as follows: (a) a determination of any disease or illness that could affect performance on various tests and/or the use of any medications for any chronic medical condition; (b) a habitual intake of Caff greater than 100 mg/day and/or a Caff allergy; (c) the consumption of any substance (such as stimulants, narcotics, or psychotropic drugs), nutritional supplements, and/or undergoing any restrictive diet control in the past three months that could have an impact on hormone levels or athletic performance; (d) the use of any form of contraception, including pills, patches, injections, implants, and intrauterine devices; and/or having any menstrual or endocrine abnormalities in the previous six months; (e) sleep issues and/or the consumption of alcohol or tobacco; and (f) less than three years of Ramadan observance.

The selection of players’ chronotypes was based on the Horne and Ostberg (1976) self-assessment questionnaires (MEQ) [34], which evaluate morningness-eveningness, because circadian typology may influence the investigation’s conclusions. The MEQ contains 19 items examining sleep and activity preferences, measured on a 4- to 5-point Likert scale between a total of 16 and 86. Subjects who displayed an extreme morning or evening type were excluded based on their responses to questions on the timing of their sleep and daily activities. Instead, all participants in this study were “neither type” chronotype, with scale scores ranging from 43 to 57. According to the Pittsburgh Sleep Quality Index (PSQI), all athletes had a normal sleep duration of 7.5 ± 0.9 h in the month before the experimental procedure [35]. In fact, it is thought that a PSQI score below 5 denotes poor sleep. All the participants that were recruited were low Caff consumers (56.2 ± 21.1 mg/d), as determined by a reliable semi-quantitative self-reported Caff intake questionnaire [36]. According to a mobile application (Mycalendar^®^; Period Tracker) that identifies the main events taking place throughout the menstruation cycle, all participants were assessed during the follicular phase and/or the luteal phase of their menstrual cycle [37].

### 2.2. Experimental Design

In order to reduce learning effects during the experiment and guarantee high-quality results, all players were familiarized with the experimental protocol and the equipment throughout the course of the two weeks before the study at two distinct times of day: 08:00 AM and 06:00 PM.

**Figure 1:**
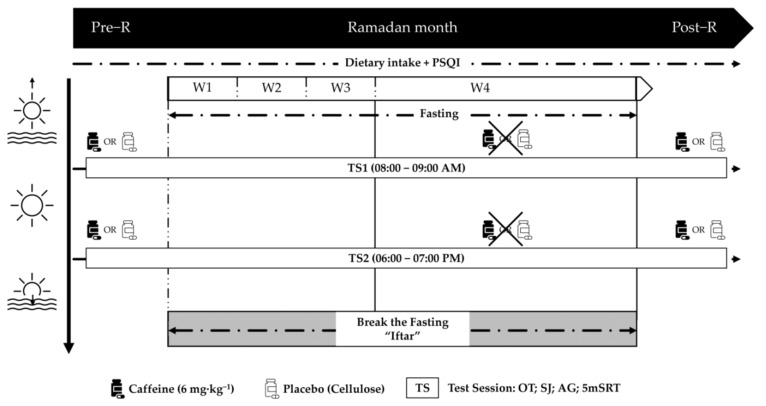
Study design: (Pre-R): the week prior to Ramadan; (R): the final week of Ramadan; (Post-R): the week immediately following Ramadan; OT: oral temperature; (SJ): squat jump test; (AG): agility test; (5 m-SRT): 5-m shuttle run test; PSQI; Pittsburgh Sleep Quality Index, all times given are expressed in local (GMT + 1 h).

The three testing periods included in the experimental protocol were: the week prior to Ramadan (Pre-R), the final week of Ramadan (R), and the week immediately following Ramadan (Post-R). Athletes completed two counterbalanced test trials throughout each period, one in the morning (AM) (08:00 AM–09:00 AM) and one in the evening (PM) (06:00 PM–07:00 PM) (Figure 1). These two times of day were chosen because they roughly refer to the batyphase and acrophase of maximum short-term performance and oral temperature [38].

Moreover, during the two periods of Ramadan ((Pre-R) and (Post-R)), each participant took part in a double-blind, randomized, and counterbalanced experimental design in two different conditions in which they ingested: (a) (Caff) (6 mg·kg^−1^) of Caffeine (BulkPowders, 100% purity; Colchester, UK); or (b) a placebo (Pla) with the same amount of an inert substance (cellulose, Guinama, Valencia, Spain) at two different times of day (08:00 AM and 06:00 PM) (Figure 1). Between sessions, at least 48 h were given for recovery, testing reproducibility, and substance washout. This dose of Caff was selected based on current literature on Caff dosages for female athletes in ball games to provide ergogenic effects on athletic performance [32,39,40]. These substances (Pla and Caff) were ingested in identical, non-identifiable capsules with 150 mL of water 60 min before the start of the experiment. In fact, a window of 60 min was chosen to allow a direct comparison with previous studies and generally reflects peak plasma concentrations of Caff after oral administration [41,42]. To ensure measurement consistency, all tests were performed on the same handball court where players train and compete, with the same testing equipment, managed by the same researcher, and in the same order. Testing was conducted at an indoor training facility with similar ambient temperatures (~25 °C, 26 °C, and 30 °C) and relative humidity (~56%, 48%, and 39%) during the three experimental periods of our study (Pre-R), (R), and (Post-R) respectively. The study was conducted in Tunisia, where Ramadan took place on May 6th and ended on 4 June 2019. During this study, the periods of daily fasting were as follows: from 03:30 AM to 07:30 PM local time (approximately 16–17 h).

### 2.3. Experimental Protocol

Once participants met all inclusion criteria and signed a parental informed consent form, they were advised to avoid all types of caffeinated food and drink (coffee, tea, chocolate, etc.) until the experiment was completed. Participants were then instructed to do the following 24 h before each experimental trial: (a) maintain their regular training load and avoid strenuous physical activity; (b) consume and replicate similar meals and drinks; (c) avoid any other stimulant substances, or Caff intake in the 24 h prior to the experiment; and (d) sleep at least 7 h the night before the test. Data from the Arabic version of the Pittsburgh Sleep Quality Index (PSQI) questionnaire were used to determine the player’s sleep parameters during the entire three test periods [35]. The weight was measured by a Tanita BC-545n (Tanita Corporation, Arlington Heights, IL, USA) to the nearest 0.1 kg. It was always performed early in the morning, with minimal clothing, no shoes, and an empty stomach. During trial days, athletes arrived at the court two hours earlier. In the experimental trials out of Ramadan ((Pre-R) and (Post-R)), participants arrived at the indoor training facility after a 6-h fasting period and therefore had a standardized breakfast for the morning trial and a light meal (~450 kcal and ~80% of CHO) for the evening trial. Following that, they ingested the corresponding capsule and rested for 60 min. However, during the Ramadan testing period (R), the fasting duration was about 4–5 h in the morning test trials and about 14–15 h in the afternoon test trials since the last meal (suhour). Only one light-to-moderate training session was performed before the first meal. During each experimental session, athletes performed the following tests after a 10 min moderate-intensity warm-up (5 min of jogging and 5-min of dynamic stretching): a squat jump test (SJ), an agility test (AG), and a 5-m shuttle run test (5 m-SRT). A 5 min rest was allowed between tests to allow adequate recovery (Figure 1).

#### 2.3.1. Dietary Intake

Additionally, during each week of the testing periods, participants’ food and drink intake in all amounts and varieties was documented in a journal. A nutritionist employed specialized software and food composition data from the (Tunisian National Institute of Statistics (1978) (https://data.bnf.fr/fr/11864527/institut_national_de_la_statistique_tunisie/?fbclid=IwAR14-99neeV572ADx3ZXqxsK46vY4KL9hPnJZQ8cBV3HPJPFt6NgzJlw3U, accessed on 29 July 2023)) to assess these results (Bilnut 4 software, Cerelles, France).

#### 2.3.2. Sleep Assessement

The Pittsburgh Sleep Quality Index (PSQI) is a 19-item questionnaire that measures sleep quality over the past month and during each experimental testing period [43]. The questionnaire assesses seven clinically derived domains of sleep difficulties (sleep quality, sleep latency, sleep duration, habitual sleep efficiency, sleep disturbances, use of sleeping medications, and daytime dysfunction). Each question is scored on a scale of 0 to 3, with higher scores indicating more severe sleep impairment. The total number of points ranges from 0 to 21. Higher scores indicate poorer sleep quality and a greater prevalence of sleep disorders. A global score greater than 5 is usually interpreted as indicating relevant sleep disturbances in at least two components or moderate difficulties in more than three components. This questionnaire was validated for the Arabic language in 2010 [35].

#### 2.3.3. Squat Jump Test (SJ)

During the SJ, participants were instructed to lower into a stationary semi-squatted position without any load at a 90° angle to the knees and hold for 2 s. They then performed a maximal vertical jump without performing a previous countermovement, as required, on an infrared jump system (Optojump, Microgate, Bolzano, Italy) interfaced with a microcomputer. We verified that no previous countermovement was used during the trial. A two-minute rest period was allowed between each of the three repetitions. For our analysis, we kept the highest jump.

#### 2.3.4. Agility Test (AG)

The Illinois agility test was administered five minutes after the SJ test. This test has been reported to be a reliable and valid test of an athlete’s ability to quickly change direction [44]. The test was conducted in a 10-by-5 m area, with four cones in the middle. Participants had one practice session. The circuit was then completed as quickly as possible. Timing gates (Brower timing systems; Draper, UT, USA) were used to record the time from start to finish.

#### 2.3.5. Five Meter Shuttles Run Test (5 m-SRT)

The test, as described in a recent study [32], consists of six repetitions of 30 s maximal shuttle sprints followed by a 10 s rest period and 35 s in between. Six beacons were placed in a straight line, 5 m apart, to cover a total distance of 25 m. Each subject began the test in line with the first beacon, then sprinted 5 m to a second beacon, touched the ground adjacent to the beacon with their hand, and returned to the first beacon, touching down on the ground adjacent to the beacon. The subject then ran 10 m to the third beacon, then back to the first, and so on until 30 s had passed. Each sprint entails running back and forth as quickly as possible over set distances of 5 m, 10 m, 15 m, 20 m, 25 m, etc., attempting to cover as much ground as possible in the 30 s [45]. During each 30 s shuttle, each subject’s distance was approximated to the nearest 2.5 m. The subjects repeated this protocol six times with a 35 s rest in between. The following parameters were calculated based on test performance:

- Total distance (TD) (m) = total distance covered during the six 30 s shuttles;
- Best distance (PD) (m) = the longest distance covered in a 30 s shuttle sprint.

### 2.4. Statistical Analysis

All statistical tests were processed with STATISTICA 10 software (StatSoft, Paris, France). Means and SD (standard deviation) values were calculated for each variable. When the Shapiro–Wilk test showed that the data were normally distributed, parametric tests were performed. For the effects of Ramadan fasting, data were analyzed using a two-way ANOVA (3 (Phases) × 2 (Time of day)) with repeated measures. For the effect of Caff, data were analyzed using a three-way ANOVA (2 (test periods) × 2 (time of day) × 2 (Caffeine)) with repeated measures. Where appropriate, significant differences between means were tested using Tukey’s HSD Post-hoc test. One-way repeated measures ANOVA (3 test periods) was used to analyze the data: PSQI, body mass, BMI, energy intake, fat (g), carbohydrate (g), and protein (g). When significant differences were reported, the Tukey Post-hoc test was used to test between means. A significant level was considered as *p*-value of ≤ 0.05. Furthermore, effect sizes (Cohen’s d) using partial eta squared (ηp^2^) were reported. Parametric effect sizes were defined as: large (≥0.14), moderate (≥0.06) or small (≥0.01) [46].

## 3. Results

### 3.1. Dietary Intake

The differences in (mean ± SD) dietary energy intake, estimated body mass, and body mass index recorded during Ramadan, as well as (Pre-R) and (Post-R), are shown in Table 2. The mean of total energy and macronutrient intake (kcal/day), dietary protein (g/d), dietary carbohydrate (g/d), and dietary fat (g/d) did not change significantly between the three testing phases (all *p* > 0.05) (Table 2). Moreover, there was no significant difference in players’ body mass or body mass index between the three testing phases. (both *p* > 0.05) (Table 2).

### 3.2. Sleep Assessement

Phases had a significant main effect on global PSQI score (F (2.24) = 94.82, *p* < 0.001, ηp^2^ = 0.88), with higher scores recorded during both Ramadan (*p* < 0.001) and (Post-R) (*p* < 0.001) compared with (Pre-R) (Table 3). After Ramadan, the overall PSQI rating was significantly higher than it was before (Pre-R) (*p* < 0.001) (Table 3). Additionally, there was a significant phases effect on sleep duration (F (2.24) = 36.53, *p* < 0.001, ηp^2^ = 0.75), quality of sleep (F (2.24) = 54.23, *p* < 0.001, ηp^2^ = 0.81), daytime dysfunction score (F (2.24) = 25.08, *p* < 0.001, ηp^2^ = 0.67), sleep disturbance score (F (2.24) = 37.41, *p* < 0.001, ηp^2^ = 0.75), and sleep efficiency (F (2.24) = 36.98, *p* < 0.001, ηp^2^ = 0.75). On the other hand, Ramadan fasting had no significant effect on the use of sleeping medication or on sleep latency.

Sleep duration declined during Ramadan (*p* < 0.001), and (Post-R) (*p* < 0.001) compared with (Pre-R), according to the post-hoc analyses. Compared with (Post-R) (*p* < 0.05) and (Pre-R) (*p* < 0.001), Ramadan phase reported poorer sleep duration. Compared with (Pre-R), the sleep quality and daytime dysfunction scores increased during both R (both *p* < 0.001) and (Post-R) (both *p* < 0.001) (Table 3). Furthermore, the subjective sleep quality scores and daytime dysfunction scores recorded in the Ramadan phase (R) were higher compared with (Post-R) (*p* < 0.05). A significant increase in the sleep disturbance scores also during Ramadan (*p* < 0.001), and (Post-R) (*p* < 0.001) as compared with BR was recorded. The sleep disturbance was higher during Ramadan compared with (Post-R) (*p* < 0.01). The daytime dysfunction score was found to increase during R (*p* < 0.001) and (Post-R) (*p* < 0.001) compared with (Pre-R) (Table 3). However, no difference was recorded in these components comparing R with (Post-R) (*p* > 0.05).

### 3.3. Squat Jump Test (SJ)

**Figure 2:**
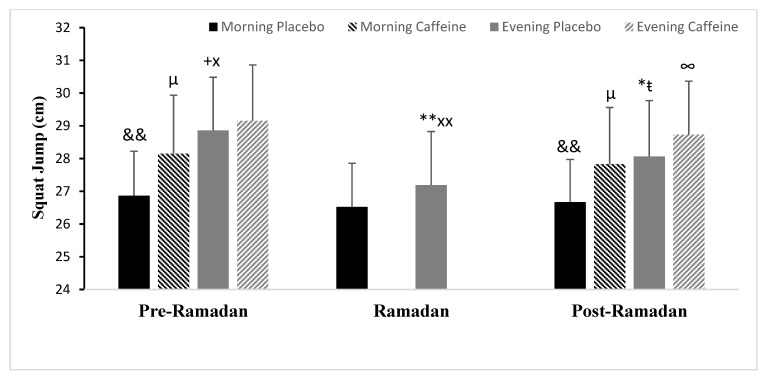
Mean ± SD values of squat jump performance (SJ) registered at 08:00 AM and 06:00 PM during Pre-Ramadan (Pre-R), Ramadan (R), and Post-Ramadan (Post-R). ^&&^ Significant difference compared with 06:00 PM (*p* < 0.05 and *p* < 0.001, respectively). *, ** Significant difference compared with (Pre-R) for the same time of the day (*p* < 0.01 and *p* < 0.001, respectively). ^ŧ^, ^+^ Significant difference compared with (R) for the same time of the day (*p* < 0.01 and *p* < 0.001, respectively). ^x^, ^xx^ Significant difference compared with (Post-R) for the same time of the day (*p* < 0.05 and *p* < 0.01, respectively). ^∞^, ^µ^ significant difference of caffeine ingestion compared with Placebo at the same time of day (*p* < 0.05 and *p* < 0.001, respectively).

Concerning Ramadan effects, the two-way ANOVA reported significant main effects of phases (F (2.24) = 33.32; *p* < 0.001; ηp^2^ = 0.73) and interaction TD (Time of Day) × Phases (F (2.24) = 68.88; *p* < 0.001; ηp^2^ = 0.85) for the SJ, but not TD. Post-hoc analysis revealed that SJ was significantly better in the evening compared with the morning (Pre-R) (*p* < 0.001). SJ performance decreased in the evening during Ramadan (*p* < 0.001) and (Post-R) (*p* < 0.05) compared with (Pre-R) (same TD), but remained constant in the morning during the three phases (same TD) (both *p* > 0.05) (Figure 2). In addition, compared with (R), (Post-R) was better (*p* < 0.01) (Figure 1).

Concerning Caff effects, there were statistically significant interactions detected between TD × Ramadan (F (1.12) =17.93; *p* < 0.01; ηp^2^ = 0.59), Caff × Ramadan (F (1.12) =13.27; *p* < 0.01; ηp^2^ = 0.52), but not between TD × Caff or between the three independent variables. The post-hoc analysis revealed that, compared with PLC, Caff improved SJ only in the morning during Pre-Ramadan and both morning and evening during Post-Ramadan. This increase was significantly larger in the morning (SJ was increased by 4.8%, *p* < 0.001) than in the evening (SJ was improved by 1%, *p* > 0.05) during Pre-Ramadan (Figure 2). During Post-Ramadan, however, this improvement was significantly stronger in the evening (SJ was increased by 2.4%, *p* < 0.05) and in the morning (SJ was increased by 4.4%, *p* < 0.001), with values approaching those of the placebo of Pre-Ramadan within the same time of day (*p* > 0.05) (Figure 2).

### 3.4. Agility Test (AG)

**Figure 3:**
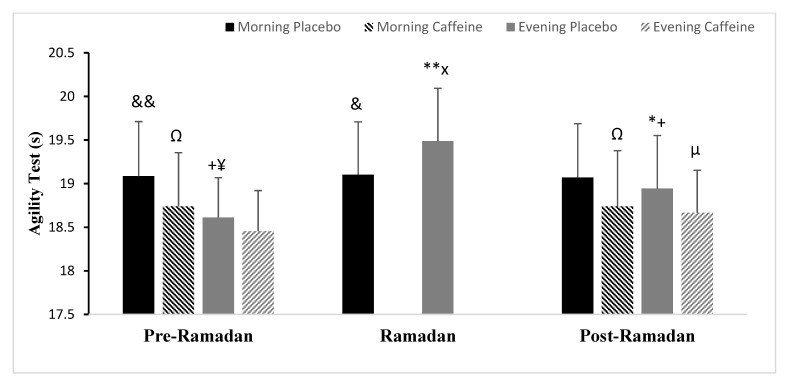
Mean ± SD values of agility test performance (AG) registered at 08:00 AM and 06:00 PM during Pre-Ramadan (Pre-R), Ramadan, and Post-Ramadan (Post-R). ^&^, ^&&^ Significant difference compared with 06:00 PM (*p* < 0.01 and *p* < 0.001, respectively). *, ** Significant difference compared with (Pre-R) for the same time of the day (*p* < 0.01, and *p* < 0.001, respectively). ^+^ Significant difference compared with (R) for the same time of the day (*p* < 0.001). ^¥^, ^x^ Significant difference compared with (Post-R) for the same time of the day (*p* < 0.01 and *p* < 0.001, respectively). ^µ^, ^Ω^ significant difference of Caffeine ingestion compared with Placebo at the same time of day (*p* < 0.01, *p* < 0.001, respectively).

In terms of Ramadan effects, the two-way ANOVA revealed that the AG had significant main effects of the interaction TD × Phases (F (2.24) =16.87; *p* < 0.001; ηp^2^ = 0.58). Post-hoc testing demonstrated that agility was significantly better in the evening than in the morning (Pre-R) (*p* < 0.001). AG performance decreased in the evening during Ramadan (*p* < 0.001) and (Post-R) (*p* < 0.01) compared with (Pre-R) (same TD), but remained constant in the morning during the three phases (same TD) (both *p* > 0.05) (Figure 3). Moreover, compared with R, (Post-R) was better (*p* < 0.001) (Figure 3).

Concerning Caff effects, there were statistically significant interactions detected only between TD × Ramadan (F (1.12) = 44.01; *p* < 0.001; ηp^2^ = 0.78). The main effects were detected for TD (F (1.12) = 34.30; *p* < 0.001; ηp^2^ = 0.74), Ramadan (F (1.12) = 26.14; *p* < 0.001; ηp^2^ = 0.68), and Caff (F (1.12) = 44.60; *p* < 0.001; ηp^2^ = 0.78). According to the post-hoc analysis, Caff improved AG in the morning and evening compared with PLC at both times. This increase was significantly larger in the morning (AG was lowered by 1.8%, *p* < 0.001) than in the evening (AG was reduced by 0.8%, *p* > 0.05) during Pre-R (Figure 3). During Post-R, however, this improvement was significantly stronger in the evening (AG was reduced by 1.7%, *p* < 0.001) than in the morning (AG was lowered by 1.5%, *p* < 0.01), with values approaching those of the placebo of Pre-Ramadan within the same time of day (*p* > 0.05) (Figure 3).

### 3.5. Five Meter-SRT

#### 3.5.1. Total Distance (TD)

**Figure 4:**
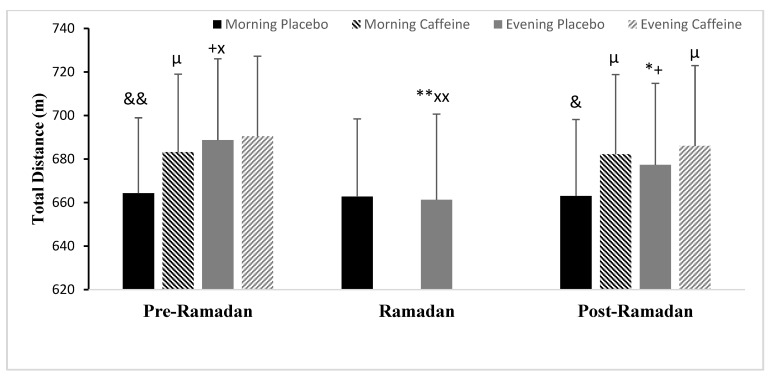
Mean ± SD values of Total distance (TD) covered during 5m-SRT registered at 08:00 AM and 06:00 PM during Pre-Ramadan (Pre-R), Ramadan, and Post-Ramadan (Post-R). ^&^, ^&&^ Significant difference compared with 06:00 PM (*p* < 0.01 and *p* < 0.001, respectively). *, ** Significant difference compared with (Pre-R) for the same time of the day (*p* < 0.01 and *p* < 0.001, respectively). ^+^ Significant difference compared with (R) for the same time of the day (*p* < 0.001). ^x^, ^xx^ Significant difference compared with (Post-R) for the same time of the day (*p* < 0.01 and *p* < 0.001, respectively). ^µ^ significant difference of Caffeine ingestion compared with Placebo at the same time of day (*p* < 0.001).

Ramadan’s observance demonstrated a significant main effect on TD × Periods interaction (F (2.24) = 41.11; *p* < 0.001; ηp^2^ = 0.77). The Post-hoc analysis revealed that TD was greater in the evening than in the afternoon (*p* < 0.001) during Pre-R. Whereas, TD performance declined in the evening during Ramadan (*p* < 0.001) and Post-R (*p* < 0.01) compared with Pre-R (same time day), it remained constant in the morning during all periods (both *p* > 0.05) (Figure 4).

Concerning Caff effects, there were statistically significant interactions detected between the three independent variables (F (2.12) = 23.05; *p* < 0.001; ηp^2^ = 0.65), TD × Ramadan (F (2.12) = 16.83; *p* < 0.01; ηp^2^ = 0.58), TD × Caff (F (2.12) = 40.89; *p* < 0.001; ηp^2^ = 0.77), and Ramadan × Caff (F (2.12) = 31.31; *p* < 0.001; ηp^2^ = 0.72). The post-hoc analysis revealed that Caff improved differently on the TD in the morning and evening, compared with PLC at both periods. This increase was significant only in the morning (TD was better by 2.8%, *p* < 0.001) and not in the evening (TD was improved by 0.3%, *p* > 0.05) during Pre-Ramadan. During Post-Ramadan, however, this improvement was recorded at both times of the day, with greater enhancement recorded in the evening. In fact, this enhancement was significantly stronger in the evening (TD was increased by 2.9%, *p* < 0.001) than in the morning (TD was increased by 1.3%, *p* < 0.001), with values approaching those of the placebo of Pre-Ramadan during the same time of day (*p* < 0.05) (Figure 4).

#### 3.5.2. Peak Distance (PD)

**Figure 5:**
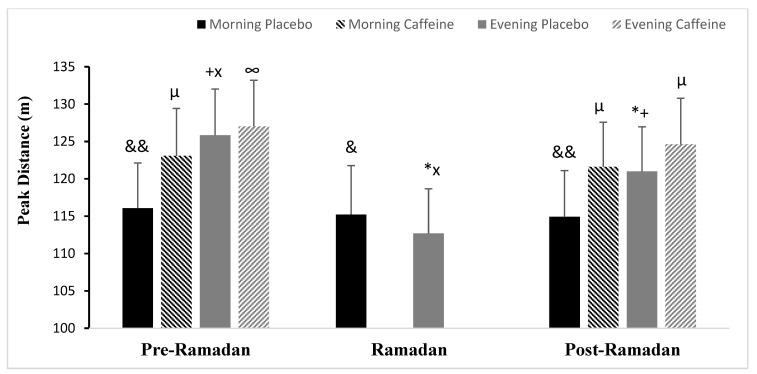
Mean ± SD values of Peak distance (PD) covered during 5m-SRT registered at 08:00 AM and 06:00 PM during Pre-Ramadan (Pre-R), Ramadan, and Post-Ramadan (Post-R). ^&^, ^&&^ Significant difference compared with 06:00 PM (*p* < 0.01 and *p* < 0.001, respectively). * Significant difference compared with (Pre-R) for the same time of the day (*p* < 0.001). ^+^ Significant difference compared with (R) for the same time of the day (*p* < 0.001). ^x^ Significant difference compared with (Post-R) for the same time of the day (*p* < 0.001). ^∞^, ^µ^ significant difference of Caffeine ingestion compared with Placebo at the same time of day (*p* < 0.05 and *p* < 0.001, respectively).

Ramadan’s observance demonstrated a significant main TD × Periods interaction (F (2.24) =184.91; *p* < 0.001; ηp^2^ = 0.93) on (PD). PD was higher in the evening than in the afternoon (*p* < 0.001) during Pre-R, according to the post-hoc analysis. PD performance decreased in the evening during Ramadan (*p* < 0.001) and Post-R (*p* < 0.001) compared with Pre-Ramadan (same time day), but remained constant in the morning during all periods (both *p* > 0.05) (Figure 5).

Concerning Caff effects, there were statistically significant interactions detected between the three independent variables (F (2.12) =19.83; *p* < 0.001; ηp^2^ = 0.62), TD × Ramadan (F (2.12) = 12.99; *p* < 0.01; ηp^2^ = 0.51), TD × Caff (F (2.12) = 23.22; *p* < 0.001; ηp^2^ = 0.65), and Ramadan × Caff (F (2.12) = 88.14; *p* < 0.001; ηp^2^ = 0.88). Caff improved PD in the morning and evening, compared with PLC during both periods. This increase was significantly larger in the morning (PD was better by 6%, *p* < 0.001) than in the evening (PD was increased by 0.9%, *p* < 0.05) during Pre-Ramadan. During Post-Ramadan, however, this improvement was significantly stronger in both the evening (TD was increased by 5.8%, *p* < 0.001) and the morning (PD was increased by 3%, *p* < 0.001), with values approaching those of the placebo of Pre-Ramadan within the same time of day (*p* < 0.05) (Figure 5).

## 4. Discussion

The current study aimed to explore the effects of pre-exercise Caff intake on high-intensity short-term physical performance (SJ, AG, and 5 m-SRT) in adolescent female handball players immediately following Ramadan. The study’s main findings were that (i) Ramadan fasting had a negative effect on short-term high-intensity physical performances (only in the evening) and sleep quality, during and immediately after Ramadan month, but not on daily energy intake or body mass; similarly, (ii) pre-exercise Caff intake reduced the negative effects of Ramadan fasting on high-intensity short-term physical performances recorded immediately after Ramadan month. Our outcomes revealed higher short-term high-intensity physical performance in the SJ test, agility, and 5m-SRT (i.e., PD and TD) performance in the evening session than the morning session before Ramadan. Several studies have shown that the best anaerobic performances were recorded in the afternoon, which is consistent with the current findings [32,47,48,49]. In this regard, [50] showed that the best time of day for any physical activity is between 04:30 PM and 06:30 PM. However, several studies [51,52,53] have not found diurnal and/or circadian fluctuations in physical performance. In fact, this daily fluctuation in performance results from the synchronization of physiological, psychological, and metabolic rhythms. All these variables attain their optimum in the early afternoon [54,55]. In this regard, the body temperature has been estimated to be 0.9% warmer in the afternoon [56], which encourages the use of glycogen rather than fats as an energy source and reinforces the interconnections among actine and myosine [57]. As a result, training or engaging in physical activity in the afternoon promotes better muscular action and power gains, even though the mechanism is not fully defined [58]. In addition to regular training time [38], differences in motivation/awakening, different chronotypes (neither chronotype in our study), and each athlete’s internal biological clock [59] may explain this effectiveness during the evening and why certain studies reveal no intraday variation.

Regarding Ramadan effects, our data indicated that Ramadan fasting (i) had a negative influence on short-term high-intensity physical performances (i.e., (SJ, AG, and 5 mRST) only in the evening, both during and immediately Post-Ramadan month; accordingly, (ii) had a negative effect on sleep quality and duration but not on athletes’ daily energy consumption and body mass. In agreement with our results, several studies reported that the various changes that occur throughout Ramadan may have an impact on athletes’ physical performance [2,60,61,62]. The current results contradict some previous studies that did not find an influence of Ramadan fasting on squat jump performance [6,7], agility capacity [8,11] or repeated sprints [3,12]. The above contradictory findings are most likely due to the assessments’ timing and protocol. The drop in performance observed only during evening sessions could be related to the prolonged fasting period, which may reduce the availability and utilization of energy substrates, and additionally affect hormonal and metabolic responses when combined with dehydration [63,64]. Caloric restriction during RF, diminished total energy and water intake, fewer daily meals (only two), and dietary changes may result in critical daytime dehydration [65], lower total protein synthesis [66], and low energy availability [67]. However, it cannot be attributed just to the increased duration of fasting observed in the evening (almost 15 h) because this impairment continued shortly after Ramadan, leading us to conclude that degradation in sleep quality can be the cause of this decline. Our PSQI results show that the average sleep loss amounted to 90 min during Ramadan and 60 min after Ramadan compared with before Ramadan. This delay was accompanied by a rise in the total PSQI score and a deterioration in sleep quality, which is consistent with a recent meta-analysis [23] and a recent study with young female handball players [2]. The amount of sleep (total sleep time), the quality of sleep, and the timing of the sleep period are all crucial elements in an athlete’s ability to train, improve training responsiveness, recover, and perform [68,69]. One probable explanation for this reduction is alterations in nocturnal sleep patterns.

Our findings regarding the effects of Caff indicate that various aspects of short-term high-intensity performance were improved following Caff consumption compared with a placebo. The present results are in contrast to previous studies on female athletes that reported no effect of Caff on jump performance [70,71,72] or repeated sprint performance [40,73,74]. However, recent studies have demonstrated that Caff consumption is a helpful supplement for jumping ability [73,75] and agility [76]. Specifically, with the same dose of Caff administered to female athletes in team sports, previous studies reported a synergistic impact of Caff on these parameters [32,39,40]. Whereas, other studies noticed no difference between placebo and Caff conditions [77,78]. Furthermore, discrepancies in variables including gender, age, time of day, fitness levels, Caff administration protocol, and discipline may justify the findings’ inconsistency. In this regard, the inhibition of adenosine receptors allows for the avoidance of neuronal activity drops and enhances muscle fiber recruitment, making it one of the most crucial mechanisms of Caff functioning [79]. Although the central nervous system is thought to be the mechanism by which Caff enhances performance [80], other mechanisms, such as myofibril augmentation, calcium availability [81,82], and energy substrate availability, have been proposed to explain Caff’s ergonomic effect [83].

On the other hand, the time of day (morning vs. evening) and Ramadan fasting appear to have an important impact on the response to Caff intake. The highest values for these variables were observed before Ramadan in the evening following Caff consumption. Caff’s effectiveness in the evening was limited before Ramadan, as a small or no significant ergogenic effect was detected in the SJ, AT, and 5 m repeated sprint tests. The results also reveal that the values of these variables following Caff administration in the morning are almost equivalent to those reported in afternoon trials without Caff supplementation. Previous studies revealed that acute consumption reversed the morning impairment in short-term high-intensity performance, allowing similar performance to that noticed in the afternoon [32,84]. These data suggest that athletes can use Caff in the morning as an ergogenic supplement to help them counteract the morning-induced decline in muscular function. These findings are mainly consistent with the current study’s findings, indicating that acute Caff consumption before exercise is an efficient ergogenic aid for contracting morning-induced deficits in short term high-intensity exercise performance.

Interestingly, we also find that, immediately after Ramadan, Caff improved performance during both the morning and evening to comparable levels to those recorded before Ramadan. However, this difference in effectiveness between before and after Ramadan is attributed to the fact that Caff productivity occurs when performance is at its lowest values, as is the case throughout this period. Moreover, it is believed that one night of partial sleep deprivation affects short-term maximal performance in the afternoon of the following day, as was the case in the study by [85]. Caffeine consumption has been shown to increase performance in the afternoon following partial sleep loss [30]. It is therefore not surprising to observe Caff productivity at both times of day during this period when sleep duration and quality have not reached their Pre-Ramadan levels.

The current investigation provides a unique addition to the literature, as Caff intake immediately following Ramadan fasting would be an effective strategy for increasing morning and evening performance when players are required to compete or exercise during the week following Ramadan. To our knowledge, no study has investigated the effects of Caff consumption at different times of day immediately following Ramadan on young female players, who, like males, have lower levels of performance during this period compared with the afternoon and who frequently participate in morning training and competitions that can coincide with Ramadan.

The strengths of the current investigation include a rigorous study design with young female athletes while examining several factors such as time of day, sleep parameters, daily caloric intake, Ramadan fasting, and several aspects of short-term high intensity performances. Aside from its strengths, some of our study’s limitations should be recognized in order to improve its application to real-life sports situations. First, the female athletes in this study were all under the age of 18, which limits the ability to apply the results to female handball players over the age of 18 and male handball players or other disciplines. Second, no blood variable data were collected during the experimental trials, ruling out any possibility that metabolic and hormonal variables play a role in Caff efficiency. Furthermore, we did not use objective methods to control the individuals’ sleep quality and amount (i.e., actimetry or polysomnography). Finally, the sample size was restricted. A large cohort study exploring the effect of Caff intake at different times of the day, in different periods (after Ramadan, during Ramadan after breaking fast, different seasons), in different conditions (after jet lag, at altitude, and in heat), in both genders, and at varying ages (e.g., adolescents and adults), should be carried out to increase the available evidence.

## 5. Conclusions

The present study showed an impairment in sleep parameters and several aspects of short-term high-intensity performance, with performance decreasing only in the evening, during, and immediately after Ramadan. Similarly, ingestion of a moderate dose of Caffeine (6 mg·kg^−1^) prior to exercise immediately after Ramadan can counteract the decline in performance observed during this period, with values close to those of before Ramadan. Caffeine’s synergistic effects might be an effective strategy for dealing with physical impairment during tournaments and competitions scheduled immediately following Ramadan, as well as for planning the pre-competitive routine or training session more successfully among young female handball players. The pre-exercise ingestion of a moderate dose of caffeine (6 mg·kg^−1)^ can bring benefits immediately after Ramadan, in both the morning and afternoon, with values similar to before Ramadan. However, our findings support the use of caffeine as an ergogenic enhancer only during morning training or competition. These guidelines are only for low-caffeine users and well-trained females who do not have an extreme chronotype.

## Figures and Tables

**Table 1:** General characteristics of the study subjects (*n* = 13).

	Minimum	Maximum	Mean
Age (years)	16	17	16.6 ± 0.5
Body mass (kg)	51	76	59.3 ± 9.1
Height (m)	1.6	1.8	1.7 ± 0.1
Body mass index (kg/m^2^)	18.3	27.5	21.4 ± 2.7
Daily caffeine intake (mg)	30	95	56.2 ± 21.1
MEQ questionnaire score (au)	43	57	50.2 ± 4.6
Handball experience (years)	3	5	3.8 ± 0.6
Training sessions frequency/week	3	5	4.2 ± 0.7
Usual training session duration (min)	60	120	90 ± 22.16

MEQ: morningness-eveningness questionnaire of Horne and Ostberg (1976) [34]. The minimum, maximum, mean, and standard deviation values of the participants’ characteristics are shown in table.

**Table 2:** Differences of means values of calories and macronutrient intake, body mass, and body mass index during three testing periods (pre-, during and Post-Ramadan).

	Pre-Ramadan	Ramadan	Post-Ramadan	*p* Value
Protein (g/d)	65 ± 15.7	62.9 ± 17.6	68.2 ± 16.2	0.31
Carbohydrate (g/d)	476.8 ± 62.5	480.6 ± 62.1	477.8 ± 61.8	0.26
Fat (g/d)	106.7 ± 18.8	107.8 ± 18.5	104.3 ± 19.6	0.12
Energy intake (kcal/day)	3282.8 ± 330.6	3300.4 ± 327.5	3277.3 ± 331.6	0.62
Body mass (kg)	59.3 ± 9.1	59.3 ± 8.6	59.58 ± 9.01	0.27
Body mass index (kg/m^2^)	21.4 ± 2.7	21.4 ± 2.5	21.5 ± 2.6	0.29

**Table 3:** Measurement of the subjective quality of sleep recorded during the week before Ramadan (Pre-R), the last week of Ramadan (R), the week after Ramadan (Post-R).

	Pre-Ramadan	Ramadan	Post-Ramadan
Sleep latency (min)	15.9 ± 1.5	16.2 ± 1.3	16.3 ± 1.3
Sleep effciency (%)	95.64 ± 4.5 ^+#^	90.03 ± 5.1 ^&^	91.67 ± 5.1 ^&^
Sleep duration (h)	7.5 ± 0.9 ^+#^	6 ± 0.7 ^&∞^	6.5 ± 0.7 ^&^*
Sleep quality (AU)	0.5 ± 0.4 ^+#^	2.1 ± 0.6 ^&∞^	1.6 ± 0.6 ^&^*
Sleep disturbances (AU)	0.5 ± 0.3 ^+#^	1.3 ± 0.4 ^&^*^Ω^	0.9± 0.2 ^&ŧ^
Daytime dysfunction (AU)	0.3 ± 0.2 ^+#^	0.7 ± 0.2 ^&^	0.9 ± 0.3 ^&^
Total score of PSQI (AU)	2.1 ± 1.2 ^+#^	6.4 ± 1.3 ^&#^	5 ± 1.2 ^+&^

^&^: Significant difference compared with (Pre-R); *, ^ŧ^, ^+^: Significant difference compared with R (*p* < 0.05, *p* < 0.01, *p* < 0.001, respectively); ^∞^, ^Ω^, ^#^: Significant difference compared with (Post-R) (*p* < 0.05, *p* < 0.01, *p* < 0.001, respectively); AU: arbitrary units; PSQI: The Pittsburgh Sleep Quality Index.

## Data Availability

The original contributions presented in the study are included in the article, further inquiries can be directed to the corresponding author.
